# Retinal ischemia following mandible tumor treatment with steroid injection

**DOI:** 10.5935/0004-2749.20200099

**Published:** 2024-02-11

**Authors:** Leonardo Lando, Hugo Mendes Silva, Lincoln Lara Cardoso, David Leonardo Cruvinel Isaac, Kim-Ir-Sen Santos Teixeira, Marcos Avila

**Affiliations:** 1 Departamento de Oftalmologia, Universidade Federal de Goiás, GO, Brazil; 2 Faculdade de Odontologia, Universidade Federal de Goiás, GO, Brazil; 3 Departamento de Radiologia e Diagnóstico por Imagem, Universidade Federal de Goiás, GO, Brazil

**Keywords:** Retina, Ischemia, Mandibular neoplasms, Jaw neoplasms, Granuloma, giant cells, Steroids, Retina, Isquemia, Neoplasias da mandíbula, Neoplasias maxilomandibulares, Granuloma de células gigantes, Esteróides

## Abstract

Central giant cell granuloma is a rare osseous tumor affecting young patients with anatomical and functional compromise of the maxilla and mandible. Steroid injection therapy constitutes a less invasive treatment modality for disease control in selected cases. Retinal ischemia is a reported complication of multiple medical procedures, including dental interventions, and may lead to loss of vision with poor prognosis. We report a case of retinal arteriolar ischemic disease following central giant cell granuloma management with local injected corticosteroids.

## Abstract (pt)

O granuloma central de células gigantes é um tumor ósseo raro que afeta pacientes jovens com comprometimento anatômico e funcional da maxila e mandíbula. A terapia com injeção de esteroides constitui uma modalidade de tratamento menos invasiva para o controle da doença em casos selecionados. A isquemia retiniana é uma complicação relatada em vários procedimentos médicos, incluindo intervenções odontológicas, e pode levar à perda da visão com mau prognóstico. Relatamos um caso de doença isquêmica arteriolar da retina após o tratamento com granuloma central de células gigantes com corticosteroides injetados locais.

## INTRODUCTION

Central giant cell granuloma (CGCG) is an infrequent tumor of the jaw that is most prevalent in young healthy patients under the age of 30 years^(1)^. CGCG exhibits benign behaviors, yet lesions may be locally invasive, requiring treatment ranging from intralesional medication therapy to surgical resection. Direct injection of corticosteroids in the lesion is a possible alternative to avoid extensive surgery for CGCG, and it allows disease control with a lower complication rate^(2)^.

Retinal arterial occlusions have been reported as a major vascular complication of multiple medical invasive interventions, including oral and maxillofacial approaches, and may lead to severe loss of vision with poor prognosis^(3^,^4)^. Complications after steroid injections in mandible granulomas are rare, and a single case in the literature describes an iatrogenic event in the eye^(1)^. We report a case of retinal ischemia following CGCG treatment with corticosteroid injection.

## CASE REPORT

**Figure 1:**
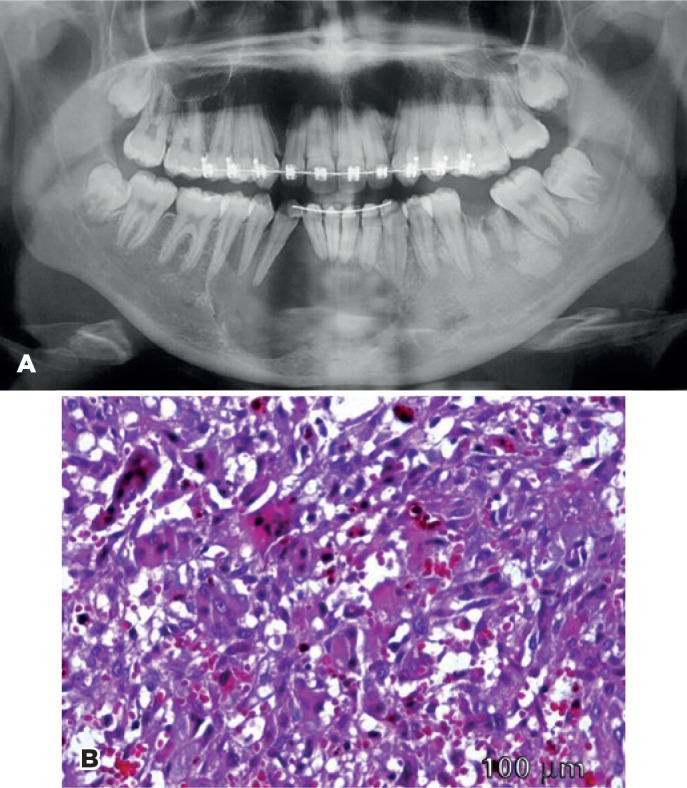
A) Radiographic imaging exhibits a radiolucent multilocular CGCG lesion in the anterior right mandible associated with tooth displacement and cortical reabsorption. B) Histological tumor analysis shows a non-encapsulated lesion with plump to ovoid cells and proliferative multinucleated giant cells in a hemorrhagic background with acute and chronic inflammation (HE-400).

A 16-year-old female with CGCG in the right mandible (Figure 1) was admitted to the ophthalmic emergency care unit for an ipsilateral acute loss of vision immediately after injection of triamcinolone acetonide (10 mg) mixed with anesthetic (2% mepivacaine) and 1:100,000 epinephrine into a local tumor. The patient sought eye evaluation within 45 minutes of visual decline, without improvement meanwhile. The patient had undergone monthly steroid injections in the jaw as part of a one-year treatment protocol, for which this would be the patient’s tenth session. The patient denied any concomitant systemic symptoms.

**Figure 2:**
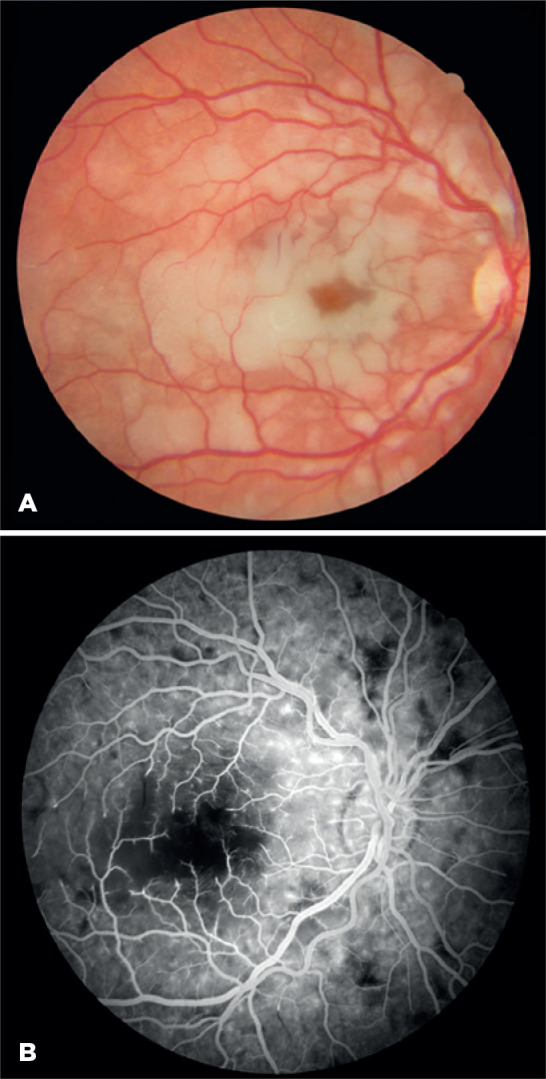
A) Right eye fundus photo displays patchy white areas of tissue necrosis at the posterior pole, resembling Purtscher retinopathy (Purtscher-like), with severe macular involvement. No local vessel obstruction is visible, yet moderate tortuosity in major arterial branches is apparent. B) Arteriovenous phase of FA in OD highlights diffuse non-perfusion of terminal arterioles with capillary loss, notably in the central macula and mid-periphery.

The patient’s best-corrected visual acuity (BCVA) was counting fingers at 1 meter in the right eye (OD) and 20/20 in the left eye (OS). The patient’s pupils were reactive, though a relative afferent pupillary defect was evident in the OD. Biomicroscopic exam and intra-ocular pressure (IOP) were normal. Fundus examination in the OD showed irregular retinal whitening at the posterior pole, diffuse vessel tortuosity, and cherry-spot macula (Figure 2A). Fundoscopy was unremarkable in the OS. Ocular massage, IOP-lowering topical drops, and anterior chamber paracentesis were promptly delivered with no improvement in vision.

Fluorescein angiography (FA, Topcon TRC 50IX; Topcon, NJ, USA) performed after primary interventions showed areas of capillary non-perfusion and macular ischemia in the OD (Figure 2B). The dye transit was normal in the OS. The patient was further evaluated by vascular and neurosurgery specialists, yet no systemic abnormality was encountered. On computer tomography (CT) angiography of the head and neck, no anomalous vascular communication was detected.

**Figure 3:**
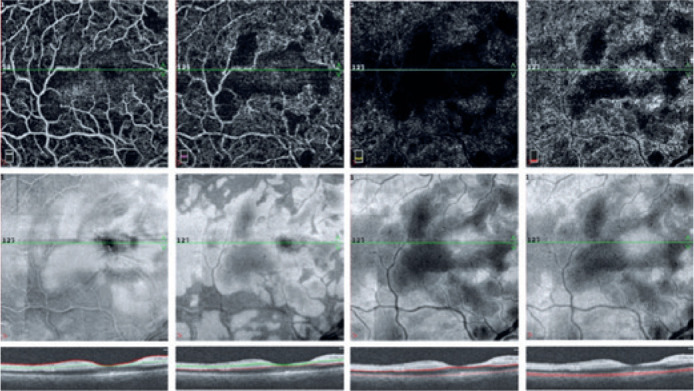
Spectral domain OCTA scans in OD one month later demonstrate reduced capillary density in all retinal layers and choriocapillaris, both in structural and *en-face* maps (automatic segmentation).

We opted for a conservative approach, and the patient was closely monitored without additional ocular intervention. Retinal damage partially recovered and BCVA was 20/100 in the OD after one month. At this moment, spectral domain OCT angiography (SD-OCTA, RTVue XR Avanti; Optovue, Fremont, CA, USA) demonstrated a decreased capillary density, including in the macular area (Figure 3). Management of the mandibular tumor was switched to surgical excision, which was performed by the oral and maxillofacial team.

## DISCUSSION

CGCG consists of benign bone neoplasms of the maxilla and mandible with an incidence of 1.1 per million^(1^,^5)^. CGCG lesions can promote bone remodeling, facial swelling, tooth displacement, and malocclusion, requiring definitive intervention^(2)^.

Treatment modalities for invasive CGCG may involve conventional surgery or alternative approaches, such as intratumoral corticosteroid injections^(5^,^6)^. Local steroid injections have been a treatment of choice for mandibular granulomas following successful strategies adopted in other solitary bone lesions with respect to safety, efficacy, and a lower degree of esthetic damage^(2)^. With this technique, multiple sites are needled, and steroid medication diluted with anesthetic is injected under high pressure at weekly intervals^(1^,^2)^. Due to disea se rarity and the low number of comparable studies, complications related to this therapy have not been well determined^(5^,^6)^.

Retinal ischemia has been recognized as a devastating complication in numerous ocular and systemic medical procedures. Vascular events may affect the central retinal artery, arteriolar branches, and small capillaries, sometimes in multiple territories, often leading to poor visual prognosis^(3^,^4)^. Two classic mechanisms are proposed to explain retinal ischemic disease, hypoperfusion and thromboembolism, such that patients should always be screened for underlying conditions if a causative factor is not immediately evident^(3^,^7)^.

A thoroughly normal systemic work-up in our patient led us to attribute the mandibular procedure as the source of retinal damage. In a similar case by Bhushan et al.^(1)^, triamcinolone crystals were observed inside the lumen of retinal vessels, elucidating an embolic basis, which was related to an anastomotic route identified on the patient’s head angiography results. Although no medication deposits were encountered in our patient’s fundus, areas of tissue pallor and arterial capillary damage noted on dye angiography confirmed typical ischemic disease.

Potential sites of anastomosis were carefully inspected on head and neck CT angiogram slices. The preserved integrity of the vasculature indicates that injection content (medication flush and/or necrotic tumor tissue) probably had penetrated the eye in a retrograde fashion and/or through physiologic collateral routes.

Arteriography studies have shown viable pathways for maxillofacial dye content to reach the eye through arteriolar branches that connect the external carotid artery, which perfuses the mandible, with the internal carotid artery, which is responsible for retinal irrigation^(8^,^9)^. Additionally, ocular complications observed after facial fillers sustain that retrograde migration, notably through facial/angular/ophthalmic arteries, might occur during the process of influx release following bolus injection^(4^,^10)^. Local pharmacologic effects of anesthetic medication seem improbable due to lack of autonomic control on the retinal microvasculature.

Treatment of retinal ischemia is limited and relies on support measures and prevention of future events^(3^,^7)^. With the lack of guidelines to manage CGCG and related complications, this particular case opens important issues regarding jaw tumor manipulation:

1. Maxillofacial specialists must be aware of potential ocular vascular complications of mandible and maxilla procedures, even local and minimally invasive ones, which would represent a lower risk of flow disturbance.
2. Patients must be preoperatively advised about risks involving any orofacial intervention with injected medication and should be closely monitored.
3. Maxillofacial protocols should be planned in accor-dance with the pressure of administration and carefully tailored to groups with a higher risk of cardiovascular complications; or patients with a single eye.
4. In the event of retinal ischemia, continuation of CGCG therapy should be weighted as a sequential occlusive episode could theoretically be facilitated by patent or newly formed collateral routes.

We recognize the limitations of this study in terms of its conclusions; therefore, further investigations are necessary to confirm our findings.
